# Retrospective space-time analysis of H5N1 Avian Influenza emergence in Thailand

**DOI:** 10.1186/1476-072X-9-3

**Published:** 2010-01-27

**Authors:** Marc Souris, Jean-Paul Gonzalez, Jothiganesh Shanmugasundaram, Victoria Corvest, Pattamaporn Kittayapong

**Affiliations:** 1 Center of Excellence for Vectors and Vector Borne Diseases, Faculty of Science, Mahidol University at Salaya, 999 Phutthamonthon 4, Nakhon Pathom 73170, Thailand; 2 UMR 190, IRD, 44, Bd de Dunkerque 13572 Marseille Cedex 02, France; 3 RS&GIS FoS, Asian Institute of Technology, PO Box 4, Klong Luang, Pathumthani 12120, Thailand; 4 Centre International de Recherches Médicales de Franceville, BP 769, Franceville, Gabon; 5 Laboratoire Espace, Santé et Territoires, Université Paris Ouest-Nanterre La Défense, 200 avenue de la République, 92001 Nanterre Cedex, France

## Abstract

**Background:**

The highly pathogenic avian influenza (HPAI) H5N1 virus remains a worldwide threat to human and animal health, while the mechanisms explaining its epizootic emergence and re-emergence in poultry are largely unknown. Data from Thailand, a country that experienced significant epidemics in poultry and has recorded suspicious cases of HPAI on a daily basis since 2004, are used here to study the process of emergence. A spatial approach is employed to describe all HPAI H5N1 virus epizootics from 2004 to 2008 and to characterize the pattern of emergence: multiple independent introductions of the virus followed by moderate local spread vs. very rare emergences followed by strong local spread and rare long range diffusion jumps. Sites where epizootics originate (by foreign introduction, local persistence, or long range jump) were selected from those to which the disease subsequently spreads using a filter based on relative date and position. The spatial distribution of these selected foci was statistically analyzed, and to differentiate environmental factors from long range diffusion, we investigate the relationship of these foci with environmental exposure factors and with rearing characteristics.

**Results:**

During each wave of epizootics, the temporal occurrence of cases did not show a temporal interruption of more than a week. All foci were globally clustered; i.e., more than 90% of cases had a previous case within a 10 km range and a 21 day period of time, showing a strong local spread. We were able to estimate 60 km as the maximum distance for the local farm to farm dissemination process. The remaining "emergent" cases have occurred randomly over Thailand and did not show specific location, clusters, or trends. We found that these foci are not statistically related to specific environmental conditions or land cover characteristics, and most of them may be interpreted as long range diffusion jumps due to commercial practices.

**Conclusion:**

We conclude that only a few foci appear to have been at the origin of each HPAI epidemic wave, leading to the practical action that surveillance and control must focus on farm to farm transmission rather than on emergence or wild fauna.

## 1. Background

World-widely spread since 2003, the Highly Pathogenic Avian Influenza (HPAI) H5N1 virus remains a major threat to human and animal health, and the mechanisms of emergence and re-emergence of avian epizootics remains poorly documented. The respective roles that are played in the emergence and spread of HPAI by agricultural and commercial practices, by wild birds, and by possible persistence of the virus in the environment, are difficult to quantify. It is an issue of research that has sparked many debates [1-6].

The association of avian influenza foci (emergent and dissemination mixed) with environmental factors has been studied for H7N1 in Italy, for H7N7 in The Netherlands, and for H7N3 in Canada and in Thailand [7-12]. Spatiotemporal characteristics of HPAI for H5N1 has been studied for Thailand and Vietnam [13,14], but there have been yet no attempts to investigate the spatiotemporal characteristics of these epizootics that might distinguish the factors associated with initial disease cases (index cases) from those associated with onward pathogen transmission and disease spread or clustering.

We used a spatiotemporal approach to define and determine the sites of emergence or reemergence in Thailand, using a follow up on HPAI reported cases in poultry by national veterinarian surveillance. In this paper, "emergence" of the disease in a farm is by definition an infection that did not come from another infected farm through a causal relationship in a process of direct local contamination. The process of emergence was separated from the dissemination through filtering procedures that used the date, location, and geographical arrangement of epizootic cases. We analyzed the spatial distribution of emergence, and the relationship between emergence, environmental conditions, and rearing characteristics.

## 2. Materials and methods

### 2.1. Data collection

**Figure 1:**
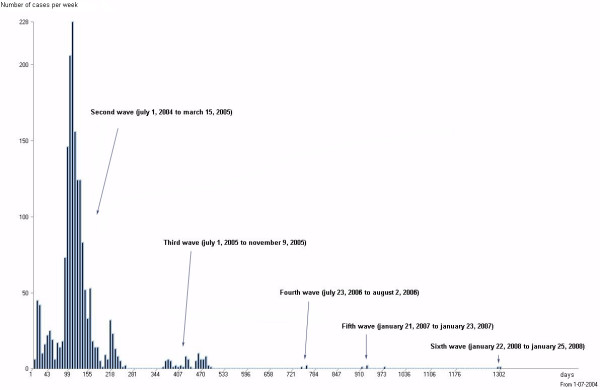
**Evolution of Avian Influenza cases number**. Histogram by week from July, 1^st^, 2004 until May, 1^st^, 2008. Six successive waves are highlighted (labeled) in the figure. The first wave (end 2003-May 2004) does not appear in the figure, as cases were recorded without enough precision.

The data used in this study were based on all the reported epizootics of H5N1 avian influenza that occurred from July 2004 to February 2008 in Thailand, which experienced significant outbreaks during 2004 and 2005 (figure 1). Our basic study units are infected farms: cases were recorded at the farm level by the Department of Livestock Development (DLD, Ministry of Agriculture and Cooperatives, Thailand), as described in previous papers [12,15]. This data correspond to the suspected cases of HPAI detected by the clinical passive and active monitoring network. The confirmatory laboratory tests were done on cloacal swabs from live poultry or from viscera of carcasses, following the protocol described by the Office International des Epizooties [16,17].

In 2007 and 2008, reports on suspected cases were still active, but since 2006 very few laboratory-confirmed cases of H5N1 have been reported. If positive but not declared H5N1 cases were present in Thailand, it follows that a major proportion of the reported cases would also be positive. Even if a statement on non-reported case number seems difficult to make, we can still estimate the probability P_+ _of a case (reported or not) to be positive. This probability can be expressed as P_+ _= 1 - e^1/n log(1-Pn)^, where Pn is the probability to have at least one H5N1 positive case among **n **cases. **n **is the sum of reported cases (**rc**) and non-reported cases (**nrc**), and **n **is unknown, but Pn can be estimated using Prc (i.e., farmers cannot usually differentiate H5N1 clinical infection from other infections, such as the Newcastle disease). In 2007 and 2008, Prc were very low, but **rc**, and so **n**, still large (as the report on cases was still active). For example, in 2007 the **rc **was 1,969 and P_+ _was near 0.

Data were incorporated into a spatial database managed by a geographic information system (GIS SavGIS). Because the exact geographical farm locations were unknown, case coordinates were assigned to the village they belong, using the village code reported in DLD files. Thailand has 72,335 villages, according to the National Statistic Office (NSO) census; every village (in rural or peri-urban areas) represent an area around a concentration of houses, but without a delimited boundary; the geographical location of a village is represented by a point, representing the geometric center of the group of houses; the mean of the minimum distance between two village centers is 1.1 km (median is 0.8 km) with a standard deviation of 0.8 km. Some analyses were conducted at the village level, using in each village the number of infected farms reported in the village. We used also the next administrative division of Thailand in sub-districts, for epidemiological mapping, for agricultural census mapping, and for environmental analysis. Sub-districts are areas with well defined boundaries; Thailand has 7,410 sub-districts, with a median surface of 50 square kilometers each, and an average centroïde minimum distance of 5.6 km (median is 5.2 km).

### 2.2. Emergence and dissemination

The mechanisms leading to a non-random spatial distribution for an epidemic of an infectious disease are divided among several factors, which can be roughly separated into two groups: initial emergence and dissemination. Initial emergence is a rare phenomenon often linked to the environment or the organization of space (e.g., land use, ecological habitat, human activity, and susceptible organisms), and sometimes linked to other rare environmental events of natural origin with highly random characteristics (e.g., meteorological events). Dissemination factors are more related to individual characteristics (e.g., susceptibility), to proximity among individuals (i.e., neighborhoods, distances, and vectors of the disease, if any), and to interactions with the environment (e.g., ecological, socio-economic, cultural, etc.) [18].

For contagious diseases, selecting emergent cases allows us to assume statistical independence between events, which then improves the power of further environmental correlation analysis. Spatial analysis was carried out to determine if the global spatial distribution of emergence deviated significantly from randomness (specific position, clusters, patterns): non-random spatial distribution of observed cases would highlight explanatory factors, while reflecting the non-random spatial distribution of environmental factors. Statistical tests of spatial analysis used to verify the global non-randomness of spatial distributions are described elsewhere [19-22]; they are mostly based on inter-event distances or nearest neighbor distances in point pattern analysis, or in studying the variability of case counts in subsets of the study region (quadrant analysis, and spatial and space time scan statistics). These tests are very discriminating in spatial pattern detection (i.e., clustered events have a very low p-value). To prevent the rejection of the null hypothesis--considering H0 as a situation which cannot be distinguished from randomness--for rare events (low p-value) that could induce emergence but should not be systematically interpreted as related to a specific location (and need to be still considered as spatially random), a risk α of 0.1% was used instead of the classical 5%.

### 2.3. Framework of Case Emergence : a filter to eliminate dissemination cases

From our definition of emergence--an infection that did not come from another infected farm through a causal relationship in a process of direct contamination--we might make the assumption that emergent events are independent, which is not the case for events in the dissemination process, where the cases are linked by direct contagion. However, this characterization is difficult to apply globally because it is impossible to determine whether a case, in the midst of others, comes from another without investigating the exact origin of infection. Therefore we adopted a more restrictive definition in order to filter emergent cases and to eliminate dissemination cases, based solely on time and distance, by defining an 'emergent' case as one in which no previous case has been detected during a period of **T **days, in a neighborhood radius of **V**.

More precisely, we assign around every case **f **a variable radius of **V**_f_, depending on the time **t:**

where **t**_f _is the infection time of case **f**.

The **V**_0 _parameter corresponds to the initial radius of potential infection of an initial case. **T **is the period of time during which the case could be regarded as infectious.

**Figure 2:**
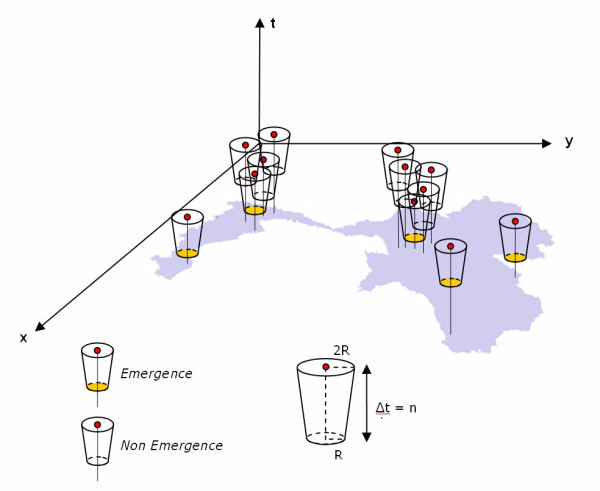
**Definition of space-time emergence**. A case is taken as 'emergent' if no other cases appear in the truncated cone defined by an "*R*" radius and a Δt height (elapsed time).

By definition, our spatio-temporal filter is constructed as follows. For a case to be 'emerging' it is necessary and sufficient that there be no other cases in the spatiotemporal truncated cones defined as (figure 2):

**f **is 'emerging' ⇔ no other case **g **different from **f **and **f **∈ D(**g**, V_g_(t_f_)),

where **D (p, V**) represents the disk of radius **V **and center **p**.

We make the radius **V **grow in a linear way during the period **T **from **V**_0 _at t_f _to 2*V_0 _at t_f_+ **T**: considering a cone and not a cylinder is an attempt to improve the method by taking into account the local spreading during the period **T**.

Applying this criterion to a set of space time point events allow us to characterize the events in two sets: emergent vs. non-emergent point events. Selecting only emergent points acts as a filter on the initial set of point events. The definition of emergence is restrictive: a real emergent case (i.e., with no relationship to the previous cases) can be considered as diffusion if it is surrounded by a previous case; cases near a previous one, in space and time, but not coming from a local diffusion process are considered as diffusion by the filter. Therefore, finding no clusters in 'emergent' cases does not mean that real emergence does not show space-time clusters. But analyzing the number and the spatial distribution of filtered cases allows us to find characteristics of real emergence. If only very few cases remain, we can conclude that the disease occurs only from very limited introductions, or that all the introductions occur at the same place and at the same time. If many cases remain, and if they are clustered, we have strong evidence that environmental factors correlate with the emergence. If many cases remain but are randomly distributed, the spatial distribution cannot help us find environmental factors; the causes of introduction may be linked to geographically random events, like anthropogenic factors.

Finding 'emergent' cases is a different problem from finding spatial or spatiotemporal clusters. An 'emergent' case that doesn't disseminate will be alone, with no cluster of cases derived from it, but an 'emergent' case that disseminates may show a posterior cluster of cases (following a contagious spatial distribution). The well-established techniques for identifying space time clusters of disease (e.g., Kulldorff's space-time scan statistics) cannot be used in emergence detection [22].

The choice of **T **(days) and **V**_0 _(radius) depends on the knowledge of biological processes and anthropogenic factors related to avian influenza (time of contagiousness, virus persistence in the environment, virus transportation, and agricultural and commercial practices). While these parameters are poorly documented or subject to high variability [23,24], we chose to study various combinations of **V**_0 _(10 to 800 km) and **T **(7, 14, 21, 28 days). Multi-testing was not an issue here, since we didn't try to find a global statistic to reject a null hypothesis.

The 21-day period following an outbreak was used for active surveillance by veterinary services in Thailand and inferred from the characteristics of the virus and the shedding of it from an infected bird. The distance appeared more subjective. When an outbreak was detected, an active surveillance involved an area within a radius of 5 km. The agricultural practices and transportation of poultry went beyond that distance, but are largely unknown and subject to high variability.

**Figure 3:**
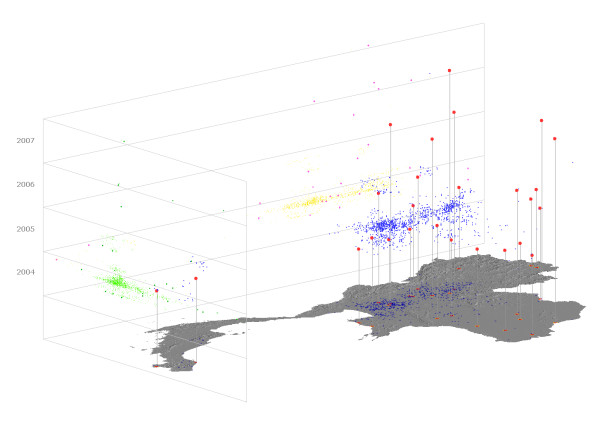
**Perspective view of HPAI cases in Thailand (28 days, 100 km)**. Vertical axis represents the time. Recorded cases are plotted in blue, and projected in green on the (longitude, time) plan, and in yellow on the (latitude, time) plan. 'Emergent' cases are plotted in red, and projected in dark green on the (longitude, time) plan and in purple in the (latitude, time) plan.

**Figure 4:**
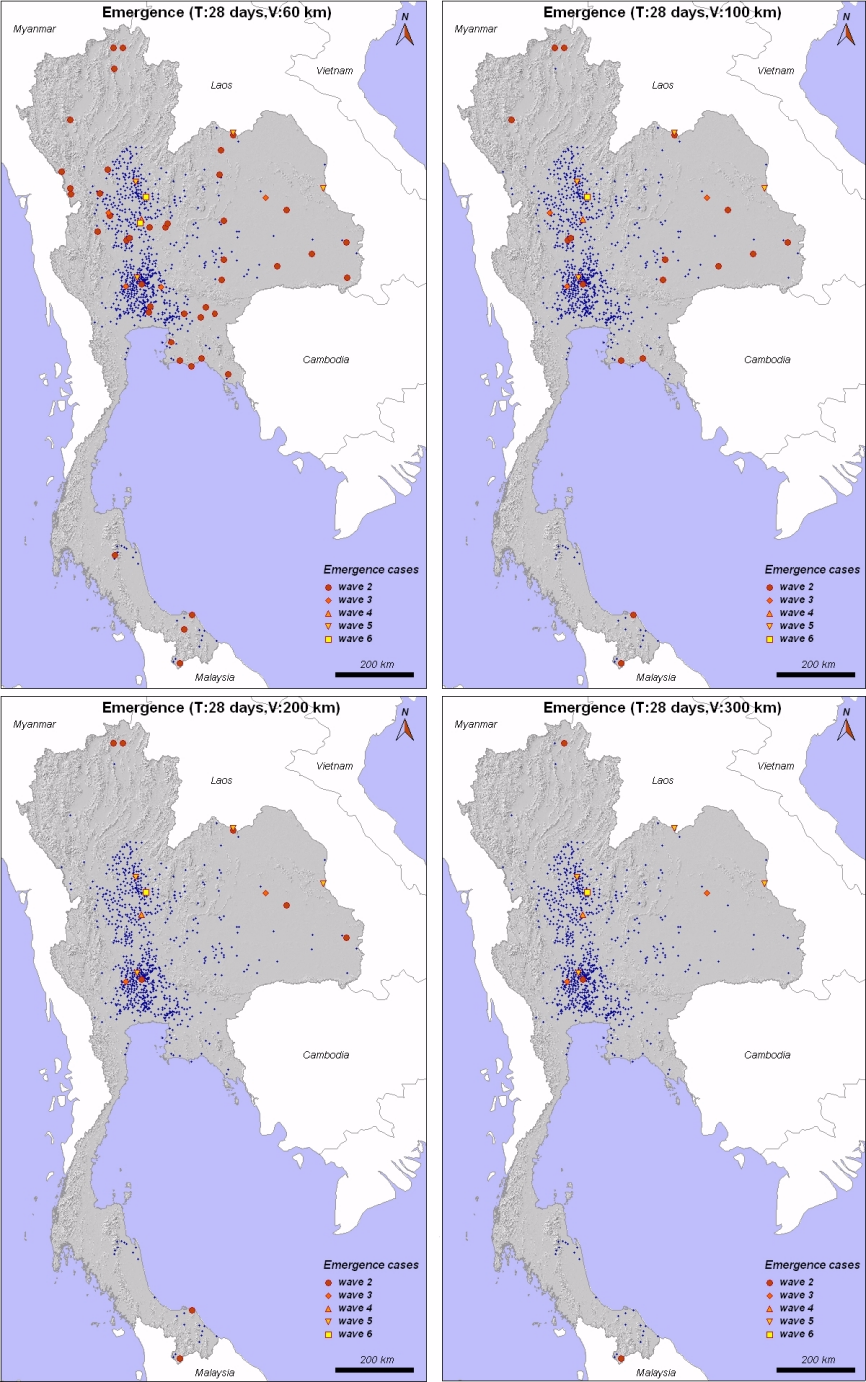
**Maps of HPAI emergence in Thailand**. "Non emergent" cases are plotted in blue.

The cases were located by the center of the village in which they belonged. The date of reporting by the DLD was potentially the date of the first clinical case at the farm and is considered in the spatiotemporal analysis. The GIS we used (SavGIS, http://www.savgis.org) allowed the selection of objects characterized with the above defined emergence criteria, and provided tools for identifying index cases (figure 3, figure 4). These space-time analysis tools were developed in the framework of this research project.

### 2.4. Geostatistical tests

Geostatistical tests were used to identify and to measure the global difference between the observed geographical distribution and a random distribution. The H0 hypothesis is always: "The spatial distribution of points was indistinguishable from a random spatial distribution" and the H1 alternative hypothesis is "the spatial distribution of points is not random". The tests, Monte Carlo type, are based on computer simulation of means or frequency of geometric indices based on the neighborhood or distance. To test whether the spatial distribution of a sub-set of points in a set of points is significantly different from random, classically, two geometric characters are used: the absolute position and the relative position of dispersion. The absolute position of a sub-set of **F **from a set of **G **can be characterized by **P**_f_, the average point (mean average of x and y) of the points F. This position is compared with the average points **P**_i _of subsets of points (with the same number of points as F) randomly selected from all points **G**, as follows: these **P**_i _means points form another cloud of points, from which we calculate the average point **P**_0_. Then we compare the distance **D**_f, 0_, between the point **P**_f _and the average point **P**_0 _of all simulated subsets, with the distribution of distances **D**_i,0_, between points **P**_i _and point **P**_0_, which have an asymptotic normal distribution, and which allow testing of hypotheses H0 and H1.

The dispersion of a sub-set **F **of points from a set **G **of points can be characterized by two indices: the average of the minimum distance between nearest neighbors in the subset **F**, and the percentage of points in the sub-set **F**, which have their nearest neighbor also in the subset **F**, the neighbors being taken from the set of points **G**. This test consists of a comparison of these two indices, calculated for the studied sub-set, with the distribution of the same indices obtained from subsets randomly selected by lottery (Monte-Carlo simulation). The MC simulation allows geostatistical tests to resolve the influence of location of points and side effects inherent to the spatial tests [19].

The list and location of all farms in Thailand is unknown, and therefore we could not perform geostatistical tests directly with reported infected farms. We performed a scale transfer from farms to villages or sub-districts, as we had villages or sub-district lists and locations for the whole of Thailand. In the scale transfer, we defined the infection as: an infected object (village or sub-district) is an object with a positive number of infected farms belonging to the object; an emergent object is an object with a positive number of emergent farms. With villages for example, we used several sets of data points: the initial set of all villages in Thailand (**G**); the subset of infected villages (**I**); and the subset of villages considered as emerging (**E**). The same calculation and characterization can be performed with sub-districts. This calculation can be performed directly in SavGIS using integration by geographical aggregation.

We applied the geostatistical tests to **I **among **G**, and **E **among **G**. All geostatistical tests were performed using SavGIS software.

### 2.5. Environmental and land cover exposure statistical tests

In order to determine the possible causes of emergence (independent introduction or long distance jumps from a previous case), we tried to find relations between emergence and environmental characteristics--mainly related to the presence of wild birds--at the village level. For land use exposure factors, we used data derived from remote sensing (Landsat-5, 30 m resolution, 2003), processed by the Land Development Department (LDD, Ministry of Agriculture and Cooperatives, Thailand). Different classes were combined to study a few major categories: wetlands; wetlands and rice fields; streams; irrigated areas; broadcasted paddy fields; transplanted paddy fields; urban areas; forests; and grasslands. With SavGIS software, we calculated for each village the surface percentage of each category in a 1 km radius around the village center; we made the assumption that the influence of environmental factors does not exceed this distance (median minimum distance between two villages centers is 0.8 km). We tested if the two groups of villages (emergent vs. non-emergent) were significantly different for each environmental category.

Due to the multiplicity of studied factors and the multi-testing problem, the risk α taken into account for each individual test is 0.5% (Bonferroni correction), and the global risk α for rejecting the null hypothesis (H0: emergent and non-emergent villages have no environmental difference) remains at 5%. We used a non-parametric test by Monte-Carlo simulations (4000), available in SavGIS software, to calculate variability and p-value for each category. We also checked the relations with agricultural and demographic data available at the sub-district level (human population density; farm density; chicken farm density; and duck farm density), using data integration of cases in sub-districts by the geo-aggregation process in SavGIS, as already described in 2.4. We used 2003 National Statistics Office census data available at the sub-district level (NSO, Ministry of Interior, Thailand). We also tested differences between emergent and non-emergent cases at the farm level using the farm characteristics reported in the DLD data (poultry type).

## 3. Results

Starting from the last trimester of 2003, several epizootic waves can be observed (figure 1). The outbreak reports of the first wave (January 2004 - April 2004) were incomplete, so our study started with the second wave (July 2004). Between July 3, 2004 and February 1, 2008, 10,319 suspected cases were reported and 1,755 have been confirmed as positive by laboratory tests. In 2007 and 2008, the suspected cases report is still active, but less than 10 reported cases have been confirmed.

### 3.1. Outbreak Mapping

Mapping at the village scale for the whole of Thailand would be difficult to read, due to the small number of cases (1,755) and the high number of villages (72,335). Therefore we performed mapping at the sub-district level after scale transfer from farm to sub-district. Mapping of infected sub-districts was done by category of infected birds, by type of farms, and by week (see maps on website: http://www.rsgis.ait.ac.th/~souris/HPAI). We also created maps using the available data on poultry farms in Thailand (NSO and DLD data - 2003). Visually, the overall distribution of all positive cases appeared not to be random in space and cases were clustered, but clusters depended on the type of bird (ducks, chicken, etc.); they also did not correspond to the spatial distribution of the farms. However, a strong visual correspondence between the spatial distribution of the cases and the density of different poultry species (especially free-range ducks) has been previously reported and statistically analyzed [12,13,25-27]. There is also a strong visual correspondence between the overall distribution of the positive cases and the distribution of farms raising layer ducks. Global geo-statistical tests confirmed that the spatial distribution of all positive cases is not randomly distributed (risk α = 0.1%), and that, as expected for a contagious disease, this spatial distribution is clustered.

### 3.2. Emergence

During each wave of epizootics, the temporal occurrence of cases did not show a temporal interruption of more than a week. More than 90% of the cases had a previous case within a 10 km range and a 21 day period of time. Applying the spatiotemporal filter eliminated most of the cases. Table 1 indicates the number of cases which can be considered as 'emerging' with relevance of **T **and **V**_0_. From a distance of 30 km and a period of time of 21 days, spatial distribution of 'emergent' cases do not show specific location, clusters, or trends; no specific geographical pattern can be highlighted (e.g.: migration corridors, wetlands, paddy fields). The selected 'emergent' cases are present over the entire territory of Thailand: no major region can be excluded from the 'emergence' process (figure 3, figure 4). Three locations within the central plain and close to the borders demonstrate repeated cases of emergence/reemergence. Geo-statistical tests show when spatial distribution of 'emergence' cannot be differentiated from random distribution (Table 2).

**Table 1:** Number of cases selected as emergent, as a function of radius distance (V) and elapsed past time without cases (T).

T	V														
	
	10	20	30	40	50	60	70	80	90	100	150	200	300	400	500
**7**	**687**	**389**	**256**	**185**	**142**	**115**	**87**	**75**	**61**	**55**	**35**	**28**	**18**	**13**	**11**
14	555	318	195	133	102	78	62	54	48	41	27	21	12	8	5
**21**	**489**	**291**	**176**	**118**	**89**	**72**	**54**	**46**	**40**	**34**	**22**	**16**	**15**	**7**	**4**
28	446	262	152	100	77	60	48	39	36	32	22	16	11	5	4
**60**	**525**	**237**	**131**	**86**	**67**	**52**	**42**	**36**	**31**	**27**	**16**	**15**	**8**	**7**	**4**
90	504	236	129	84	68	52	42	33	31	27	16	14	8	7	4

Parameters values are expressed as: T = time by days and V = distance in kilometers.

**Table 2:** Geo-statistical test for randomness, using the minimum distance between neighbor cases of same value (risk α = 0.1%).

T	V														
	
	10	20	30	40	50	60	70	80	90	100	150	200	300	400	500
**7**	**H1**	**H1**	**H1**	**H1**	**H1**	**H0**	**H0**	**H0**	**H0**	**H0**	**H0**	**H0**	**H0**	**H0**	**H0**
14	H1	H1	H1	H1	H0	H0	H0	H0	H0	H0	H0	H0	H0	H0	H0
**21**	**H1**	**H1**	**H1**	**H0**	**H0**	**H0**	**H0**	**H0**	**H0**	**H0**	**H0**	**H0**	**H0**	**H0**	**H0**
28	H1	H1	H1	H0	H0	H0	H0	H0	H0	H0	H0	H0	H0	H0	H0

Parameter values are as in Table 1 (T and V); H0 = null hypothesis (cannot be differentiated from a random pattern); H1 = alternative hypothesis (non random pattern).

### 3.3. Environmental and anthropogenic exposure factors

The statistical results of tests conducted on environmental exposure ('emergent villages' compared to 'other villages') show that if we keep the risk α at 0.5% for each factor, no environmental factor or land cover characteristic is statistically associated with emergence (for a risk α at 5%, only the values of the percentage of broadcasted paddy field area are significantly different - p = 0.02).

On the other hand, poultry type (from DLD classification) is significantly different between emergent and non-emergent farms (Table 3): egg chicken farms and chicken farms are more related to emergence (11% vs. 4.5% and 9.5% vs. 4.9%), but duck farms, especially egg duck farms, less so (4.7% vs. 15%). If we exclude from emergence possible long-distance (less than 300 km) diffusion jumps (**V**_0 _= 300 km, **T **= 21 days, Table 4), we find that francolin farms (quails) are at high risk for emergence (p-value = 0.02%).

**Table 3:** Exposure factor: type of poultry in emergent vs. non-emergent farms (V = 60 km, T = 21 days, 72 farms)

Type of poultry	Emergent farms (%)	Non-Emergent farms (%)	p-value (%)
Batam Cock	1.6	0.5 ± 0.9	15 (H0)

Domestic Chicken	59.2	55.3 ± 6.2	24 (H0)

Domestic Duck	1.6	1.1 ± 1.3	36 (H0)

Egg Chicken	10.9	4.4 ± 2.5	0.5 (H1)

Egg Duck	4.7	15.6 ± 4.2	0.7 (H1)

Farm Chicken	9.4	4.9 ± 2.6	4.9 (H1)

Farm Duck	3.2	7.6 ± 3	8.1 (H0)

Farm Francolin	3.2	2.2 ± 1.8	30 (H0)

Fighting Cock	3.2	2.7 ± 2	42 (H0)

Free range duck	1.6	1.1 ± 1.5	47 (H0)

Goose	0	0.8 ± 1	23 (H0)

Mandarin Duck	1.6	2.5 ± 1.9	30 (H0)

Turkey	0	0.2 ± 0.6	34 (H0)

**Table 4:** Exposure factor: type of poultry in emergent vs. non-emergent farms (V = 300 km, T = 21 days, 12 farms).

Type of poultry	Emergent farms (%)	Non-Emergent farms (%)	p-value (%)
Batam Cock	8 (1 farm)	0.5 ± 2	0.003 (H1)

Domestic Chicken	42 (4 farms)	55.3 ± 14	16 (H0)

Domestic Duck	0	1.1 ± 3	35 (H0)

Egg Chicken	16 (2 farms)	4.4 ± 6	2 (H1)

Egg Duck	8 (1 farm)	15.6 ± 10	25 (H0)

Farm Chicken	0	4.9 ± 6	20 (H0)

Farm Duck	0	7.6 ± 7	15 (H0)

Farm Francolin	16 (2 farms)	2.2 ± 4	0.02 (H1)

Fighting Cock	0	2.7 ± 5	27 (H0)

Free range duck	0	1.6 ± 4	32 (H0)

Goose	0	0.8 ± 2	38 (H0)

Mandarin Duck	8 (1 farm)	2.5 ± 4	10 (H0)

Turkey	0	0.2 ± 1	43 (H0)

## 4. Discussion and conclusion

### 4.1. Limitations

Our definition of emergence is sensitive to the omission of a positive case declaration, but as mentioned above and in previous studies (§2.1), the case report from July 2004 is robust and Thailand is recognized to have a good veterinarian surveillance system [12]. Our definition is also largely conservative; i.e., some cases may have been discarded, though they could have been associated with previous emergence. As noted, the choice of **V**_0 _and **T **depends on biological or anthropogenic parameters which are poorly known (e.g., virus persistency and a variety of agricultural and commercial practices). Due to these limitations, this study must be interpreted as an attempt to model emergence rather than fully reflect the reality of the epidemics, which will never been known.

### 4.2. Analysis

Several studies have shown that migratory birds are able to exchange influenza A viruses and transport them over long distances [28-33]. The role of migration among certain species of the family Anatidae in spreading the H5N1 subtype already has been suggested, but it appears from the study of this literature that no certainty can be advanced about the long-distance spread of the virus by migration [34-36]. Agro-commercial activities have been identified as major factors of local dispersion of the virus. Backyard poultry, which are extensively present everywhere in Thailand and beyond most measures of bio-security, can promote the maintenance and local spread of the disease. Free grazing ducks have been identified as an important risk factor in the spread of the virus from wild to domestic birds and between farms [12]. Poultry market activities (including poultry staying overnight in the markets or unsold poultry returned the farm) have been highlighted as a main cause of amplification and spread in some countries [27,37], but this is not a concern in Thailand, as the country has only few live-bird markets.

All these epidemiological risk factors involve short distances in the dissemination process (a few tens of kilometers). In our geo-statistical analysis, a general trend clearly separates the two situations, **H0 **and **H1**, as a function of **V**_0 _and **T **(Table 2). This trend and partition into two groups can be interpreted as supporting the existence of a limit to dissemination by proximity, and which allows us to estimate 60 km as the maximum distance for the local dissemination process. With these parameters (21 days, 60 km), most of the cases have been eliminated by our spatiotemporal filter, while 72 cases remain highlighted as 'emergent'. These cases have three possible origins: new introductions from external sources (i.e., cases in other countries; migratory birds); environmental emergence or re-emergence by local virus persistence (in soil, in water [24], or in a possible still unknown animal reservoir); long distance jumps from previous cases by agro-commercial practices or wild (resident or migratory) bird movements. Increasing the radius of exclusion in the spatiotemporal filter allows us to discard the possible long distances jumps. From 300 km for **V**_0 _(radius of exclusion), a limited number of cases (5 to 11) are considered as emergent, disregarding the chosen time of exclusion; thus with these hypotheses, it is likely that the epidemics of each identified wave came from a very limited number of original sources. The geographical location of these cases does not show any clustering; and we observed repeated cases at the borders which reinforce the hypothesis of human introduction by cross-border trade of poultry.

This result brought us, therefore, to investigate direct causes of infection in the cases that fall between these two dissemination situations (60 km/300 km)--which represent about 60 cases (among a total of 1,755 cases)--and to focus our attention on anthropogenic (agro-commercial practices) or environmental (wild birds, persistence) factors for these cases. Statistical results show they are significantly (risk α = 1%) more related to chickens than other kinds of livestock, and that environmental conditions (presence of water, water as physical vector, farm density, constructed areas, land use characteristics, and population density) are not significantly different. It is then reasonable to conclude that most of these 60 cases may be interpreted as diffusion jumps and are probably related to low frequency human practices that encourage the spread over these distances (i.e., interprovincial rearing practices: the purchase of chickens and the sale of egg laying hens; and game practices, such as fighting cocks).

### 4.3. Conclusion

In conclusion, our findings suggest that only a few index cases are responsible for each HPAI epidemic wave and that no geographical locations or environmental conditions can be highlighted in the risk of introduction of HPAI. Control need therefore be focused on dissemination rather than on emergence, in order to avoid local farm to farm transmission, medium or long distance jumps caused by agro-commercial practices, and introduction caused by cross-border trade of poultry.

## Competing interests

The authors declare that they have no competing interests.

## Funding

This project was funded by ANR (The French Agency for National Research) as part of the ECOFLU program (ANR 06SEST12, p.i. Prof. Patrick Potier, Lyon I University, France).

## Authors' contributions

MS designed the study, carried out an extensive analysis of the data, developed software, performed the interpretation, and prepared the draft of the manuscript; JPG interpreted the data, revised the manuscript and provided intellectual discussions; JS and VC contributed substantially on the data acquisition, data mapping, and the process for data analysis; PK provided intensive review of the manuscript and gave a final approval for the manuscript to be submitted for publication. All authors edited and commented on the manuscript.
